# Robotic placement of experimental prototypes for wireless gastric electrical stimulation

**DOI:** 10.1007/s00423-025-03834-1

**Published:** 2025-08-12

**Authors:** Jonas F. Schiemer, Karen Stumm, Yves Olsommer, Hauke Lang, Nadine Baumgart, Jan Baumgart, Werner Kneist

**Affiliations:** 1 Department of General, Visceral and Transplant Surgery, University Medical Center of the Johannes Gutenberg-University Mainz, Langenbeckstreet 1, Mainz, 55131 Germany; 2 Department of General and Visceral Surgery, St. Josefs-Hospital Wiesbaden, Beethovenstreet 20, Wiesbaden, 65189 Germany; 3 Translational Animal Research Center, University Medical Center of the Johannes Gutenberg-University, Hanns-Dieter-Hüsch- Weg 19, Mainz, 55128 Germany; 4 Department of Biomedical Engineering, Fraunhofer Institute for Biomedical Engineering, Joseph-von-Fraunhofer-Weg 1, 66280 Sulzbach, Saar Germany; 5 Department of Molecular and Cellular Biotechnology/Nanotechnology, Saarland University, Gustav-Kirchhoff-Street 2, Ilmenau, Saarbrücken, 98693 Germany; 6 Department of General, Visceral and Thoracic Surgery, Klinikum Darmstadt, Grafenstreet 9, Darmstadt, 64283 Germany

**Keywords:** Robotic surgery, Experimental surgery, Gastroparesis, Motility disorders, Electric stimulation, Electromyography, Implants, Medical devices

## Abstract

**Introduction:**

Gastric electrical stimulation (GES) is an effective treatment for gastroparesis. However, the available devices are equipped with bulky batteries that need to be replaced regularly by surgery.

**Methods:**

Our new implantable system consists of only 6 passive components in addition to a diode and does not require a battery. Two acute porcine experiments were carried out with a robotic surgical system for placement of the prototypes. The stimulation parameters were set with an extracorporeal unit and GES was performed. The recorded electromyography (EMG) signal was subjected to a multiresolution analysis.

**Results:**

The robot-assisted placement of the prototypes was successful. The inductive energy transfer was confirmed to be functional and EMG analysis revealed changes in gastric electrical activity.

**Conclusions:**

Further technological and rapid regulatory solutions are being sought in order to start a clinical trial with the next generation devices in the near future.

**Supplementary Information:**

The online version contains supplementary material available at 10.1007/s00423-025-03834-1.

## Introduction

Gastroparesis (GP) is a motility disorder caused by abnormal gastric myoelectric activity, disrupting slow wave frequency and amplitude. The clinically available gastric electrical stimulation (GES) device has been proven effective in alleviating GP symptoms [1, 2]. However, this device relies on a non-rechargeable battery with a limited lifespan of 5–10 years, necessitating surgical replacement once depleted [3]. 

To address these challenges, the “INTAKT project” aims to develop wireless implants for treating motility disorders throughout the gastrointestinal (GI) tract [4, 5].

## Methods

### Anaesthesia and surgery

Acute experiments were performed on two healthy pigs (40 and 46 kg) under general anesthesia following approved animal care protocols (State Office for Occupational Safety, State of Brandenburg; business reference 2347-30-2019). After overnight fasting, pre-anesthesia was administered using ketamine, midazolam, and detomidine, with general anesthesia maintained via ketamine and midazolam. Pancuronium was used for muscle relaxation, and ventilation was initiated after tracheotomy. Euthanasia was performed with 20 ml of 7.45% KCl.

**Fig. 1:**
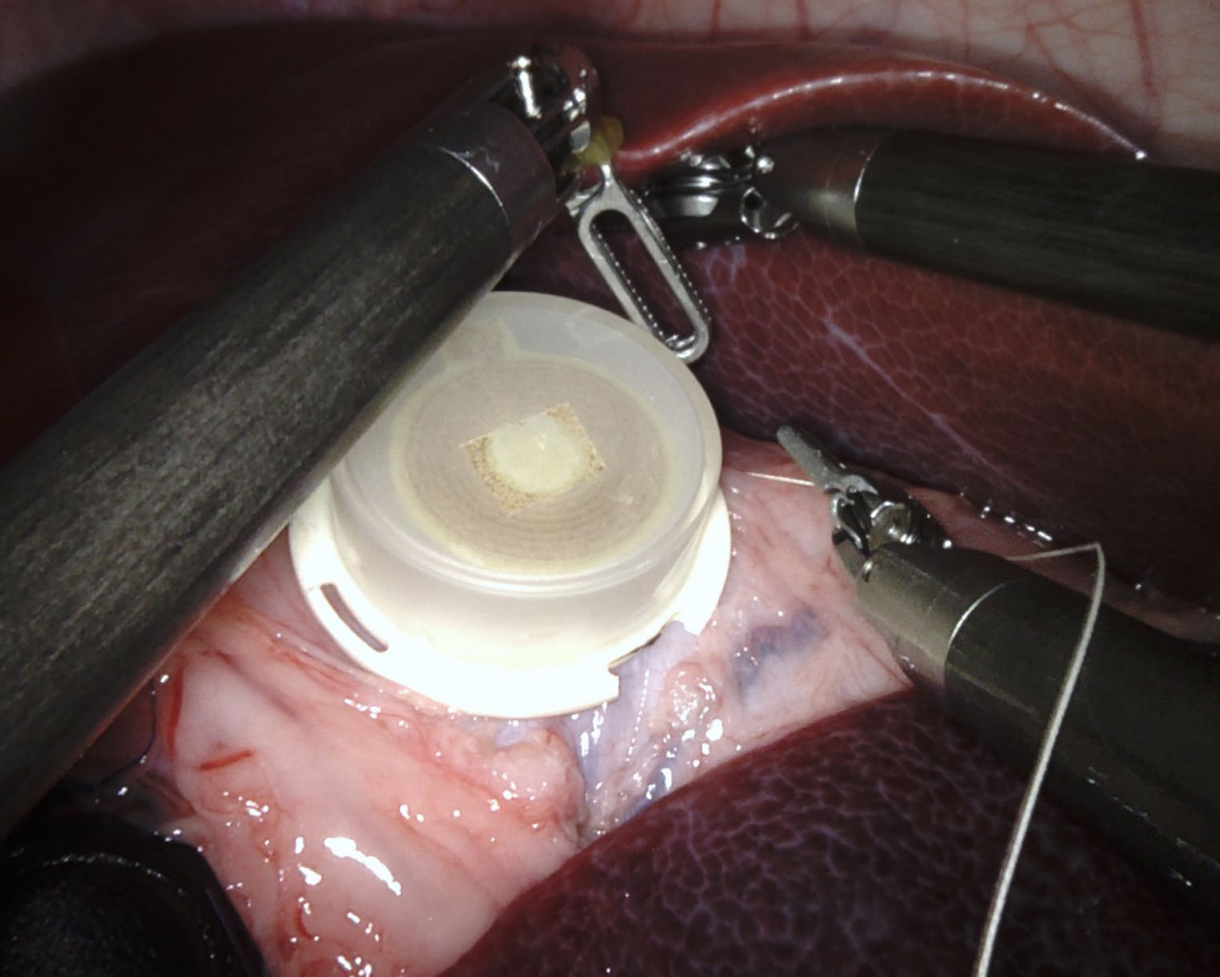
Intraoperative view of the prototype device positioned on the stomach using robotic instruments

A 10 mmHg capno-pneumoperitoneum was created, four robotic ports were inserted, and the da Vinci Xi robotic system was docked. Hook electrodes were positioned on the stomach in a standardized manner. The prototype implant was inserted via a small Alexis wound retractor and secured to the stomach’s serosal surface with three-point seromuscular sutures (Fig. 1, Supplemental Video).

Smooth muscle electromyography (EMG) was recorded using bipolar hook electrodes, with impedance measured before baseline recording (20 kHz, 10 mVpp; 0.5 Hz–2 kHz filters, ISIS Xpress system, inomed Medizintechnik GmbH, Emmendingen, Germany). The gastric prototype then wirelessly delivered 30-second inductive stimulation (20–50 Hz, 200 µs pulse), followed by 90 s of post-stimulation EMG recording.

### Prototype

The implantable device is enclosed in a hermetically sealed Perlucor ceramic cylinder (CeramTec GmbH, Plochingen, Germany), designed to accommodate a minimal set of passive and active electronic components. A set of eight circularly arranged electrodes is integrated into a cermet base plate, which also includes three suture eyelets to enable mechanical fixation during surgical implantation. Wireless power transfer is achieved via a 16 mm diameter induction coil embedded within the ceramic housing.

Power is delivered inductively at a carrier frequency of 9.5 MHz using a 50 mm transmit coil in the extracorporeal unit. The implanted receive coil is part of a parallel resonant circuit, tuned to match the carrier frequency to maximize voltage gain across the coil. The induced voltage is rectified using a diode-based rectifier and subsequently smoothed by a capacitor. This rectified voltage supplies the stimulation circuitry. Stimulation pulses are generated by discharging the storage capacitor across selected electrode pairs through a resistor-capacitor compensation network. The stimulation output consists of capacitively charge-balanced monophasic stimulation pulses. Pulse timing parameters, including frequency and duration, are modulated externally by amplitude modulation of the power carrier signal from the extracorporeal unit.

The implant functions solely as a voltage source; it does not contain current-regulating circuitry, active pulse shaping elements, or feedback systems. Moreover, it lacks integrated communication or telemetry interfaces, and thus no real-time monitoring of stimulation parameters or physiological signals is possible. Due to the absence of feedback, the exact stimulation current and tissue impedance remain unknown during operation.

**Fig. 2:**
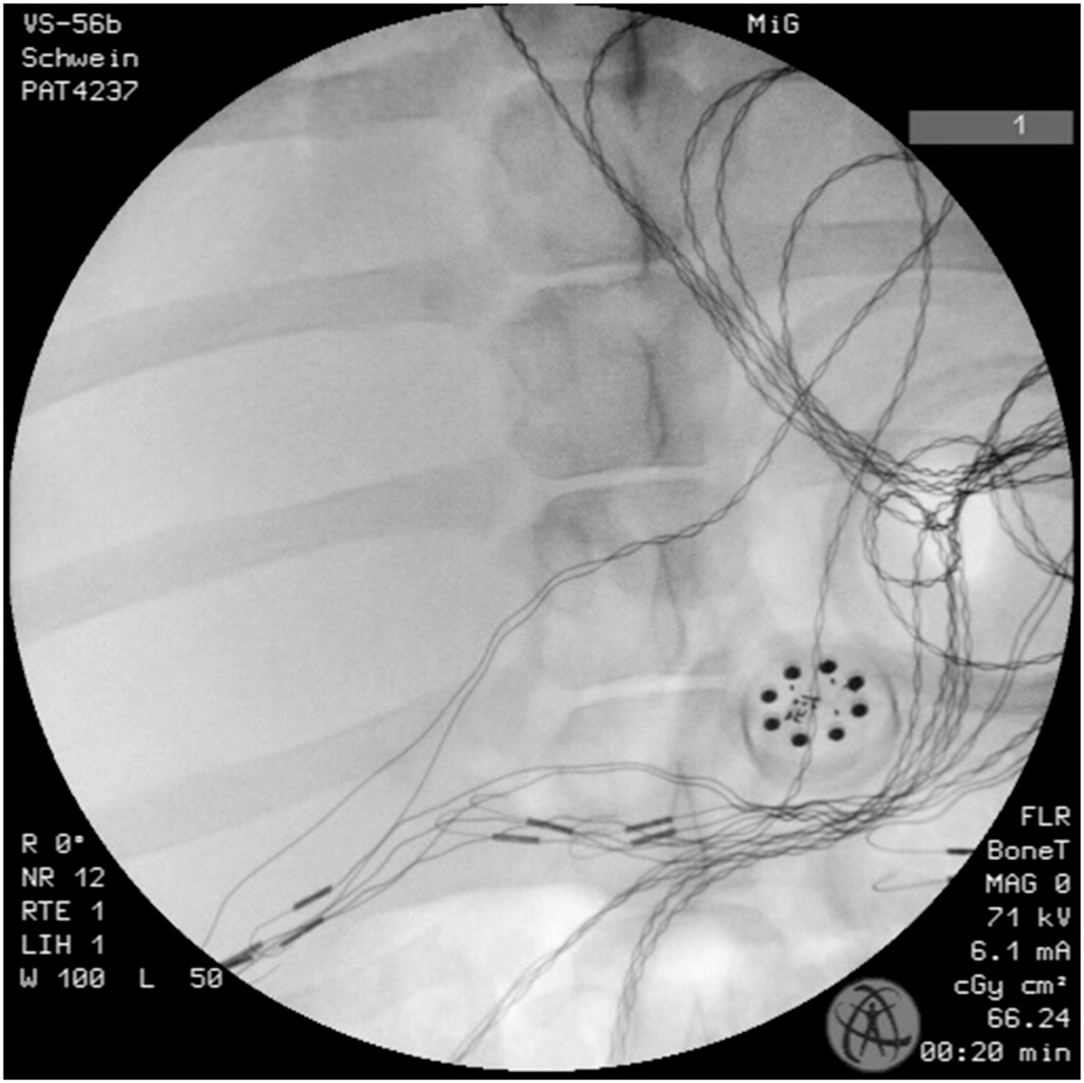
Conventional X-ray image shows the prototype positioned on the stomach. The leads of the hook electrodes for the EMG recordings are also visible

Laparoscopic diaphanoscopy was used to optimize implant positioning for maximum energy transfer, followed by radiological verification (Fig. 2). The distance between the two coils was between 30 and 50 mm.

### EMG analysis

To analyze the resulting EMG data, Maximal Overlap Discrete Wavelet Transform (MODWT) analysis was applied. This technique is a fundamental analytical construction in wavelet theory that describes the approximation properties of the discrete wavelet transform, which is ideal for biosignals that are typically discontinuous and non-periodic. The MODWT works without downsampling (no signal loss due to reduction of the sampling rate) and decomposes the signal into different frequency components. The overlapping windows in the MODWT result in more robust noise suppression and improved detection of weak or short-term signal events. We choose to decompose the EMG signals into 12 frequency band components. Among them, detail level 6 was selected for further analysis as it corresponded to the frequency range of interest for gastric slow waves (frequency band 1.53–3.18 Hz). Within level 10, the cumulative electrical activity, the area under the curve (AUC), was computed for 30-second analysis windows (frequency band 0.1–0.2 Hz.

## Results

There were no anesthesia- or surgery-related complications. The robot-assisted procedure ensured safe implant placement without perforation, significant blood loss, dislocation, or rotation.

Although baseline activity varied, both frequencies increased AUC during stimulation, reaching 263 mVs (20 Hz) and 316 mVs (50 Hz) (Table 1). Post-stimulation, AUC at 20 Hz declined steadily to 168 mVs (60–90 s), returning to baseline. At 50 Hz, AUC fluctuated, initially dropping to 172 mVs (0–30 s), rising to 277 mVs (30–60 s), then slightly decreasing to 246 mVs (60–90 s).

**Table 1:** Smooth-muscle electromyogram (EMG) analysis of two acute Porcine experiments. After recording a baseline EMG, each stomach was wirelessly stimulated (Stim.) with the prototype implant for 30 s with 20 or 50 hz and pulse wave of 200 µs. Following each stimulation event, post-stimulation EMGs were recorded over a period of 90 s. To analyze the resulting 41 EMG recordings, multiresolution analysis was applied for the calculation of cumulative electrical activity, the area under the curve (AUC), and of mean slow wave cycles per minute

Frequency [Hz]	Pulse wave [µs]	Pre-Stim. 30 s	Stim. 30 s	Post-Stim. 0–30 s	Post-Stim. 30–60 s	Post-Stim. 60–90 s
Area under the curve [mVs]						
20	200	142	263	227	190	168
50	200	187	316	172	277	246
Slow wave cycles per minute [cpm]						
20	200	3	3	2	1	1
50	200	1	4	3	2	2

Slow wave rates increased during stimulation, remaining at 3 cpm (20 Hz) and rising to 4 cpm (50 Hz). Post-stimulation, 20 Hz showed a steady decline to 1 cpm (30–90 s), while 50 Hz remained higher, stabilizing at 3 cpm (0–30 s) and decreasing to 2 cpm (30–90 s).

Higher frequencies (50 Hz) induced stronger and more sustained gastric responses, while lower frequencies (20 Hz) produced a stable, gradual return to baseline.

## Conclusion

This study presents a proof-of-concept for a wireless GES device powered via inductive energy transfer. A key feature of the system is its simplified electronic design, which does not require internal communication modules, firmware, or a battery, thereby enabling further miniaturisation and potentially enhancing robustness and long-term reliability. In acute porcine experiments, the device was implanted using a robotic surgical approach and was able to modulate gastric myoelectric activity through externally controlled stimulation parameters. Further studies are required to evaluate the chronic functionality, long-term safety, and therapeutic efficacy of the novel GES system.

Tables:

Table 1.

## Supplementary Information

Below is the link to the electronic supplementary material.

Supplementary Material 1

## Data Availability

No datasets were generated or analysed during the current study.
